# CD90^+^ Stromal Cells are Non-Professional Innate Immune Effectors of the Human Colonic Mucosa

**DOI:** 10.3389/fimmu.2013.00307

**Published:** 2013-09-30

**Authors:** Benjamin M. J. Owens, Tessa A. M. Steevels, Michael Dudek, David Walcott, Mei-Yi Sun, Alice Mayer, Philip Allan, Alison Simmons

**Affiliations:** ^1^Translational Gastroenterology Unit, Nuffield Department of Medicine, Experimental Medicine Division, John Radcliffe Hospital, University of Oxford, Oxford, UK; ^2^MRC Human Immunology Unit, John Radcliffe Hospital, Weatherall Institute of Molecular Medicine, University of Oxford, Oxford, UK

**Keywords:** stromal cells, mucosal immunology, innate immunity, intestinal homeostasis

## Abstract

Immune responses at the intestinal mucosa must allow for host protection whilst simultaneously avoiding inappropriate inflammation. Although much work has focused on the innate immune functionality of hematopoietic immune cells, non-hematopoietic cell populations – including epithelial and stromal cells – are now recognized as playing a key role in innate defense at this site. In this study we examined the innate immune capacity of primary human intestinal stromal cells (iSCs). CD90^+^ iSCs isolated from human colonic mucosa expressed a wide array of innate immune receptors and functionally responded to stimulation with bacterial ligands. iSCs also sensed infection with live *Salmonella typhimurium*, rapidly expressing IL-1 family cytokines via a RIPK2/p38MAPK-dependent signaling process. In addition to responding to innate immune triggers, primary iSCs exhibited a capacity for bacterial uptake, phagocytosis, and antigen processing, although to a lesser extent than professional APCs. Thus CD90^+^ iSCs represent an abundant population of “non-professional” innate immune effector cells of the human colonic mucosa and likely play an important adjunctive role in host defense and immune regulation at this site.

## Introduction

Emerging data suggests that non-hematopoietic stromal cells of the intestine exhibit a wider array of immunological functions than has been hitherto attributed to them. This includes the production of and response to cytokines, functional interactions with hematopoietic immune cells, the regulation of immune cell localization, and cell-intrinsic innate immune functions (1, 2). As data from both human (3) and murine (4) systems suggested that intestinal stromal cells (iSCs) express functional PRR pathways, we sought to better define the contribution of iSCs to the innate immune mechanisms operating in the human gut. Here we extend the characterization of primary stromal cells isolated from the human colonic mucosa, focusing on their ability to contribute to cell-intrinsic innate immune responses to an invasive mucosal pathogen. We confirm previous observations that human iSCs express Toll-like receptors (TLRs), and the NOD-like receptor (NLR) NOD2 at the mRNA level, and reveal that primary iSCs also express multiple other members of the NLR family of PRRs. We further show that human iSCs express detectable protein levels of NOD2 and can respond to the bacterial component muramyl dipeptide (MDP) at a functional level. Similar to observations in other cell types including myeloid dendritic cells (DCs) and a macrophage cell line (5, 6), NOD2 triggering synergizes with TLR2 stimulation to increase the magnitude of the transcriptional response to isolated bacterial components. We show that the expression of multiple IL-1 family member cytokines is regulated by PRR triggering in primary iSCs, with a particularly strong response to flagellin. Consistent with this, iSCs internalize and respond rapidly to *in vitro* infection with the flagellated bacterium *Salmonella typhimurium*, increasing their expression of IL-1β and IL-33 at both the mRNA and protein level, with this sensing dependent on RIPK2/p38MAPK signaling. In addition to these potent cell-intrinsic innate sensing responses, primary iSCs had some phagocytic and antigen-processing potential, although this was limited when compared to professional APCs. Thus we identify CD90^+^ stromal cells as “non-professional” innate immune effector cells of the human colonic mucosa.

## Materials and Methods

### Intestinal tissue preparation

Human colonic tissue specimens from macroscopically healthy areas of colon were collected from colorectal cancer patients undergoing surgery. All patients were recruited from the Translational Gastroenterology Unit and the colorectal surgery department at the John Radcliffe Hospital, Oxford. Ethical approval was obtained from the Oxfordshire Research Ethics Committee (reference number 11/YH/0020), and all study participants gave informed written consent. Colonic tissue was collagenase-digested and processed as previously described (7), and cells from the 30–40% Percoll gradient interface used for subsequent stromal cell staining and culture.

### Intestinal stromal cell culture

Cells were isolated from Percoll gradients and washed in PBS containing 2% Bovine Serum Albumin (BSA). Cells were resuspended at 1 × 10^6^ cells/ml in complete DMEM (cDMEM) containing 10% FCS, l-glutamine, 10,000 U/ml Penicillin and Streptomycin, 0.025 μg/ml Amphotericin B, 10 μg/ml Ciprofloxacin and 40 μg/ml Gentamycin (Sigma Aldrich, UK). Cells were initially plated in six well tissue culture plates (5 × 10^6^ cells/well) or T25 culture flasks (15 × 10^6^ cells/flask). After 48–72 h, non-adherent cells were removed by vigorous washing and media replaced with fresh cDMEM. Cultures were monitored for the appearance of highly adherent stromal cells with typical fibroblastic morphology. When confluent, stromal cells were harvested using 0.1% Trypsin-EDTA (Sigma Aldrich) and split at ratios of 1:3–1:5. Stromal cultures were expanded in T75 flasks and all experiments were performed using stromal cells between culture passages 3–8.

### PRR stimulations and bacterial infections

For stromal cell PRR stimulations, 50,000 iSCs/well were plated in flat-bottomed 96 well plates. After 24 h, media was changed and cells were stimulated with combinations of the following PRR ligands: 1 μg/ml PAM_3_CSK_4_, 10 μg/ml MDP, 100 pg/ml Flagellin (Invivogen, USA). At 24 h post-stimulation, cells were lysed for subsequent RNA analysis. For bacterial infections, GFP – *Salmonella enterica typhimurium* strain 12023 (kind gift from David Holden) was grown to logarithmic phase (OD less than 0.5) in LB containing ampicillin at 37°C. Infections were performed on 50,000 stromal cells/well in cDMEM without antibiotics at a multiplicity of infection (MOI) of 10 using live bacteria or bacteria killed by heating to 65°C for 60 min. For short-term infection experiments, cells were lysed for RNA extraction at 60 and 120 min post-infection. For 24-h infection experiments, 10 μg/ml Gentamycin was added to all cultures at 4 h post-infection to prevent bacterial overgrowth, and cell supernatants harvested at 24 h post-infection. Proteins were quantified in cell culture supernatants using DuoSet ELISA kits according to manufacturers instructions (R&D Systems, UK). Where indicated, RIPK2/p38MAPK signaling was inhibited using 10 μM SB203580 (in DMSO – Sigma Aldrich), and control cells were treated with DMSO alone.

### Monocyte isolation and dendritic cell generation

CD14^+^ Monocytes were purified from peripheral blood mononuclear cells of healthy donors using CD14 MACS beads (Miltenyi Biotec, Germany) as previously described (8). For transcriptional profiling, monocytes were immediately washed, lysed in RLT buffer (Qiagen) containing 1% β-Mercaptoethanol (Sigma Aldrich) and frozen for subsequent RNA extraction (RNeasy Mini Kit, Qiagen). To generate DCs, CD14^+^ monocytes were cultured for 5 days in DMEM containing 10% FCS in the presence of recombinant human GM-CSF and IL-4 (PeproTech, UK), as previously described (8).

### Flow cytometry

To assess surface protein expression by flow cytometry, cells isolated directly *ex vivo* or cells cultured *in vitro* were washed in PBS 2% BSA, and labeled for 30 min on ice with eFluor^780^ viability stain (eBioscience, San Diego, CA, USA) and anti-human monoclonal antibodies against EpCAM (Clone 9C4), CD45 (Clone HI30), and CD90 (Clone 5E10, PerCP-Cy5.5 – all BioLegend, San Diego, CA, USA) for stromal cells, or eFluor^780^ viability stain and anti-CD11c (Clone 3.9 – eBioscience) for DCs. Cells were washed twice in PBS/BSA and fixed in BD Cytofix solution. For assessment of intracellular expression of NOD2, stromal cells were surface stained as before, fixed and permeablized using BD Cytofix/Cytoperm. Cells were subsequently stained for 30 min on ice with anti-human NOD2 (Clone 2D9 – Cayman Chemical, USA) or appropriate isotype control. Cells were washed twice with BD Perm buffer and labeled for 30 min on ice with AlexaFluor^647^-conjugated anti-mouse IgG (Life Technologies, UK) followed by one wash in BD Perm buffer and a final wash in PBS/BSA. Samples were acquired using a BD LSRII and data analysis performed using FlowJo Software (Treestar, USA).

### Stromal cell Phosflow™

Intestinal stromal cells were labeled for 30 min on ice with eFluor^780^ and anti-human CD90 as previously described. After washing iSCs were plated in 150 μl cDMEM in 96 well plates at 100,000 stromal cells/well and rested at 37°C for 60 min. Cells were subsequently stimulated in sequence with 10 μg/ml MDP for various time periods, or remained unstimulated. After 60 min, cells from each stimulation condition were fixed simultaneously for 15 min at 37°C with an equal volume of pre-warmed BD Cytofix buffer. Cells were transferred to v-bottomed 96 well plates and subsequently washed in chilled PBS/BSA. Cells were permeablized using BD Phosflow Perm Buffer IV for 30 min at room temperature in the dark. Cells were washed twice in PBS/BSA and subsequently labeled with anti-human Phospho-RIP2 (Ser 176 – Cell Signaling Technology, USA) or appropriate rabbit isotype control in 50 μl PBS/BSA for 60 min at room temperature. Cells were washed and subsequently labeled with AlexaFluor^647^-conjugated anti-rabbit IgG (Cell Signaling Technology) for 30 min on ice and washed twice in PBS/BSA. Samples were acquired using a BD LSRII and data analysis performed using FlowJo Software (Treestar, USA)

### Determining bacterial uptake by fluorescence and confocal microscopy

Myeloid dendritic cells (mDC) and iSC were seeded at 1 × 10^5^ cells per well onto eight-well poly-d-lysine-coated Tissue Culture Slides (BD Biosciences). Cells were allowed to adhere for 2 h before addition of GFP-*Salmonella typhimurium* for a further 2 h (MOI = 30). Following this incubation, cells were washed with PBS and fixed in 4% paraformaldehyde for 10 min at RT. Cells were then permeabilized with 0.1% (vol/vol) Triton X-100 in PBS. Cover slips were mounted using Vectashield Mounting Medium with DAPI (Vector Laboratories). A Zeiss Qimaging Retiga 2000R was used for immunofluorescence microscopy. For confocal microscopy, iSCs were seeded at 0.5 × 10^5^ cells per well onto eight-well poly-d-lysine-coated PCA Slides (Sarstedt Ltd.). Cells were allowed to adhere overnight before addition of GFP-*Salmonella enterica typhimurium* for 4 h (MOI = 100). Following this incubation, cells were washed with PBS and fixed in 4% paraformaldehyde for 10 min at RT. Cells were then permeabilized with 0.1% (vol/vol) Triton X-100 in PBS and incubated with AlexaFluor^647^-conjugated anti-human Vimentin XP^®^ Monoclonal Antibody (Cell Signaling Technology) in PBS-1% BSA for 30 min at RT. After staining, cover slips were mounted using Vectashield Mounting Medium with DAPI (Vector Laboratories). Images were acquired using a Zeiss 780 inverted confocal microscope with an Objective Plan-Apochromat 63×/1.40 Oil DIC. During acquisition a pinhole size of 0.9 μm was used.

### qRT-PCR

For transcriptional analysis cells were lysed in RLT buffer (Qiagen) containing 1% β-Mercaptoethanol (Sigma Aldrich). Samples were homogenized using QIAshredder columns and RNA extracted using RNAeasy spin technology, according to manufacturers instructions (Qiagen). cDNA was synthesized from isolated RNA using a High Capacity cDNA Reverse Transcription Kit, according to manufacturers instructions (Life Technologies, USA). cDNA was used for qRT-PCR reactions with buffers and specific primer-probe sets from the TaqMan system (Life Technologies). In all cases *GAPDH* was used as an endogenous reference gene, and changes in expression levels calculated using either 2^∧^ − Δ*C_t_* to give levels of expression as arbitrary units (AU) relative to *GAPDH* or 2^∧^ − ΔΔ*C_t_* to give the fold increase in expression in experimental vs. control (unstimulated) conditions.

### Antigen processing and phagocytosis assays

For antigen processing and phagocytosis assays, Day 5 mDCs or cultured iSCs were plated at 100,000 cells/well in 24 well plates and pulsed with 100 μg/ml DQ-OVA (Invitrogen) or Phagocytosis assay reagent (Cayman Chemicals) for 4 h. Cells were pulsed at either 37°C or on ice. Subsequently, cells were stained for flow cytometry as previously described. Antigen processing/bead uptake was assessed as the fold increase in FITC-expression by cells at 37°C over background processing/uptake levels determined in CD11c^+^ mDCs or CD90^+^ iSCs after pulsing with the respective reagents on ice. Flow cytometry data was acquired and analyzed as previously described.

### Statistical analysis

All analysis was performed in GraphPad Prism (v5.0d). For comparisons between two groups an unpaired Student’s *t*-test was used; for comparisons across multiple groups either a one-way ANOVA, followed by a Bonferroni post-test, or a Kruskal–Wallis, with Dunn’s post-test, were performed. *p* values of less than 0.05 were considered significant.

## Results

### CD90^+^ colonic stromal cells express diverse PRRs and functional NOD2

**Figure 1:**
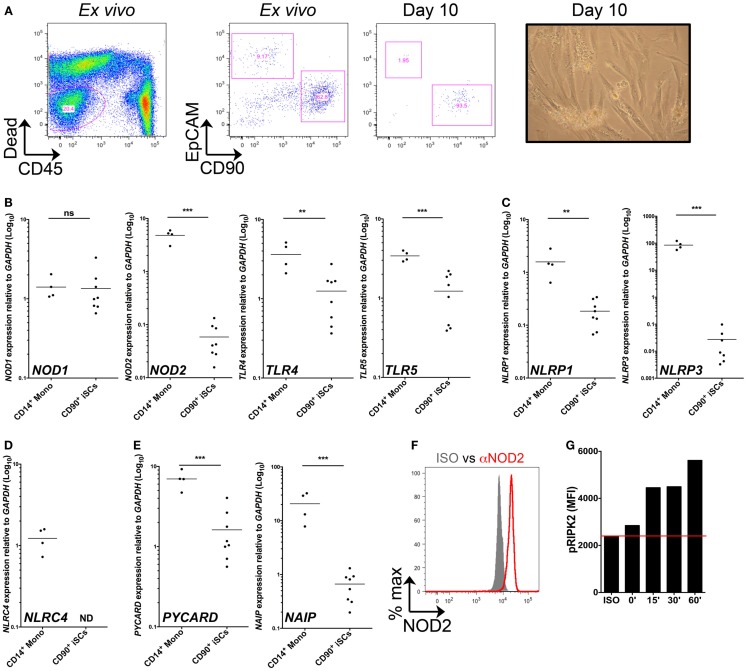
**CD90^+^ colonic stromal cells express diverse PRRs and functional NOD2**. **(A)** Shows representative flow cytometric staining of collagenase-digested colonic tissue *ex vivo* and cultured stromal cells after 10 days in culture, revealing iSCs as viable, CD45^−^EpCAM^−^CD90^+^ cells; and a representative image of day 10 cultured stromal cells obtained by light microscopy. **(B–E)** Show quantification of the expression of the indicted genes in monocytes and cultured iSCs by qRT-PCR, expressed as arbitrary units (AU) relative to *GAPDH*. **(F)** Shows quantification of NOD2 expression by intracellular flow cytometry; gray filled histogram indicates isotype control, red open histogram indicting specific staining. **(G)** Shows accumulation of phosphorylated RIPK2 in cultured iSCs determined by flow cytometry after stimulation for the indicated time periods with MDP. Data in **(A)** are representative of tissue, **(B–E)** are from monocytes isolated from *n* = 4 healthy peripheral blood donors and iSCs cultured from colonic tissue of *n* = 8 uninflamed surgical patients. Data in **(F,G)** are from iSCs of one patient, representative of four patients with similar results. **p < 0.01, ***p < 0.001.

We first sought to characterize PRR expression by stromal cells of the human colonic mucosa. Collagenase-digested colonic mucosal tissue contained an abundant population of viable, CD45^−^ non-hematopoietic cells (Figure 1A). When assessed by flow cytometry directly *ex vivo*, this population comprised EpCAM^+^ epithelial cells, CD90^+^ stromal cells, and a group of EpCAM^−^CD90^−^ non-hematopoietic cells, likely containing populations of endothelial and other stromal cell types (2). After 10 days of culture, most cells were negative for both CD45 and EpCAM, but expressed CD90 and had a fibroblastic morphology, similar to the features of iSCs previously characterized in colonic tissues of mice and humans (9, 10) (Figure 1A). Upon continued culture the cells retained CD90 expression throughout multiple culture passages *in vitro* (data not shown). Thus CD90^+^ stromal cells represent a culturable population of primary non-hematopoietic cells in the human colon. We next compared levels of PRR expression in iSCs to a prototypic “professional” innate immune cell population. CD14^+^ monocytes were purified from peripheral blood of healthy donors. The levels of PRR mRNAs in monocytes and iSCs were quantified by qPCR as AU relative to the housekeeping gene *GAPDH*. Consistent with previous reports from human and murine systems (3, 4), iSCs expressed a wide array of PRRs, although in general expression levels were lower than that in blood monocytes (Figures 1B–E). Uniquely amongst the PRRs examined, the expression of *NOD1* in iSCs was equivalent to that in CD14^+^ monocytes, at 1.41 ± 0.45 vs. 1.35 ± 0.87 AU (*p* = ns), respectively (Figure 1B). In contrast *NOD2* was expressed at significantly lower levels in iSCs than monocytes (0.06 ± 0.04 vs. 4.80 ± 1.26 AU, *p* < 0.001) and *TLR4* expression was similarly lower in iSCs (1.25 ± 0.82 vs. 3.61 ± 1.43, *p* < 0.01) (Figure 1B). iSCs also expressed *TLR5*, albeit at lower levels than monocytes (Figure 1B). iSCs additionally expressed mRNA for the inflammasome-linked sensors *NLRP1* and *NLRP3* (Figure 1C), although expression was significantly lower compared to monocytes. Although previous data has indicated that murine stromal cells express abundant levels of NLRP6 (11), expression of *NLRP6* mRNA in human iSCs was very low and not detectable in cells isolated from all patients (data not shown). Unlike monocytes, iSCs did not express detectable levels of the inflammasome sensor *NLRC4* (Figure 1D) together indicating that iSCs express specific members of PRR families, and not low levels of all receptors. As inflammasome assembly and function is critically dependent on the adapter protein Asc (*PYCARD* in humans) (12) we sought to determine whether *PYCARD* expression was detectable in iSCs. Similar to the NLR inflammasome sensors themselves, iSCs did express *PYCARD* mRNA, but at significantly lower levels than monocytes (Figure 1E). Interestingly the NLR co-receptor *NAIP* was consistently expressed in iSCs, although also at significantly reduced levels when compared to monocytes (0.66 ± 0.38 vs. 20.62 ± 11.98 AU, *p* < 0.001).

Given that mRNA expression of *NOD2* was so much lower than in monocytes, we next sought to determine whether iSCs expressed detectable protein levels of NOD2 and whether this expression allowed them to respond to the prototypic NOD2 ligand, Muramyl dipeptide (MDP). When assessed by intracellular flow cytometry we found that viable cultured CD90^+^ stromal cells expressed NOD2 at the protein level (Figure 1F). This was functional, since upon stimulation with MDP *in vitro*, expression of phosphorylated RIPK2 by iSCs increased in a time-dependent fashion, with levels up to threefold higher after 60 min of stimulation with MDP (Figure 1G). Taken together, the expression profiling and functional stimulation experiments suggest that iSCs are equipped with multiple PRR-linked innate sensing mechanisms and can respond to a bacterial PRR ligand that activates NOD2.

### Bacterial ligands regulate IL-1 family member cytokine expression in iSCs

**Figure 2:**
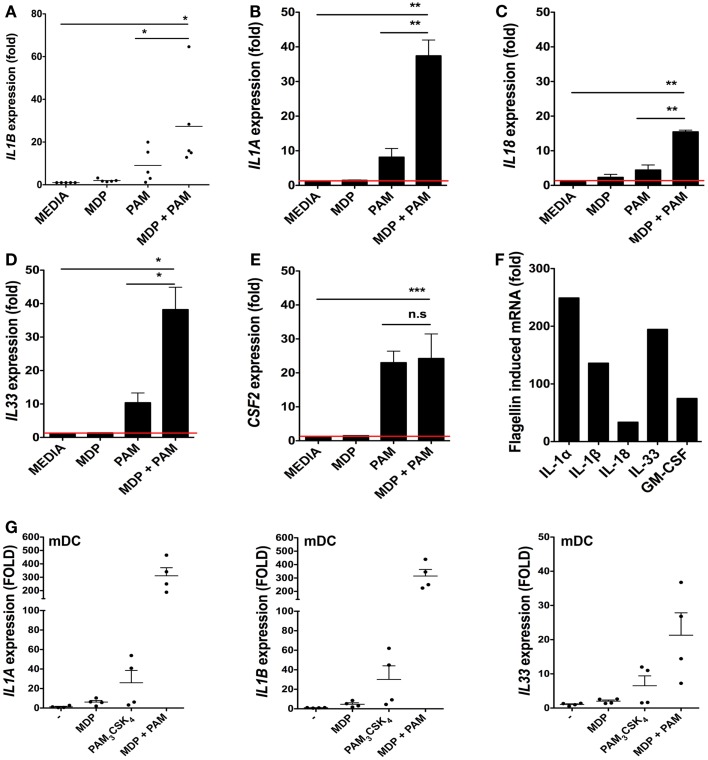
**Bacterial ligands regulate IL-1 family member cytokine expression in iSCs**. **(A–F)** Show changes in expression of the indicated cytokines by cultured iSCs, **(G)** shows the same by monocyte-derived dendritic cells (mDCs), determined by qRT-PCR after 24 h of *in vitro* stimulation with the PRR ligands indicated. Data in **(A–E)** show mean fold changes ±SEM in iSCs from colonic tissue of *n* = 5 surgical patients, **(F)** shows the mean induction of expression of the indicated genes by iSCs from one patient after stimulation with flagellin, representative of three experiments with similar results. **(G)** Shows mean fold changes ±SEM in expression of the indicated mRNA in stimulated mDCs generated from *n* = 4 peripheral blood donors. *p < 0.05, **p < 0.01, ***p < 0.001.

We next determined the extent to which isolated bacterial components could regulate cytokine responses at the transcriptional level in iSCs. As the IL-1 family is involved in mucosal homeostasis we focused on IL-1 family cytokines. We stimulated primary CD90^+^ iSCs *in vitro* with bacterially derived PRR ligands and determined the regulation of cytokine mRNA expression (Figure 2). As bacterial triggering through NOD2 synergizes with TLR2 signaling for maximal functional responses in myeloid cells (8), we initially sought to determine whether similar synergy also existed in iSCs. Culture of iSCs with MDP alone induced limited upregulation of *IL1B* mRNA expression (Figure 2A). In contrast, triggering of TLR2 in iSCs with the TLR2 agonist PAM_3_CSK_4_ resulted in an ∼eightfold increase in *IL1B* expression. Combined triggering of NOD2 and TLR2 significantly enhanced expression of *IL1B* mRNA, to levels ∼24-fold higher than in unstimulated cells, which was significantly higher than either NOD2 or TLR2 triggering in isolation. This NOD2/TLR2 synergy in the activation of cytokine transcription was also extended to several other IL-1 family members; with combined MDP and PAM_3_CSK_4_ stimulation leading to significantly enhanced levels of *IL1A* (*p* < 0.01 Figure 2B), *IL18* (*p* < 0.01 Figure 2C) and *IL33* (*p* < 0.05 Figure 2D) mRNA expression than either ligand in isolation. However this synergy was not extended to *CSF2* (GM-CSF; granulocyte-monocyte colony stimulating factor) expression, where combined NOD2/TLR2 triggering regulated mRNA for this cytokine at similar levels to TLR2 triggering in isolation (Figure 2E).

As iSCs were found to express TLR5 at the mRNA level (Figure 1B), we sought to determine the impact of stimulation with the TLR5 ligand flagellin on cytokine transcription (Figure 2F). Stimulation of iSCs with flagellin led to a dramatic increase in mRNA encoding IL-1α, IL-1β, IL-18, IL-33, and GM-CSF, with increases in expression of all cytokines ∼two to sixfold higher than combined stimulation via NOD2/TLR2. When compared directly to TLR/NLR ligand stimulated mDCs, iSCs showed a more modest fold increase in *IL1A* and *IL1B* mRNA levels, particularly apparent with dual MDP and PAM_3_CSK_4_ triggering, although a similar fold increase in IL-33 mRNA transcription was evident between the cell types (Figure 2G). Therefore iSCs can respond to TLR2 and NOD2 ligands with a similar functional synergy to that seen in other immune cell types – including DCs – but also respond strongly to stimulation with extracellular bacterial flagellin.

### Rapid internalization and sensing of live *Salmonella* by iSCs

**Figure 3:**
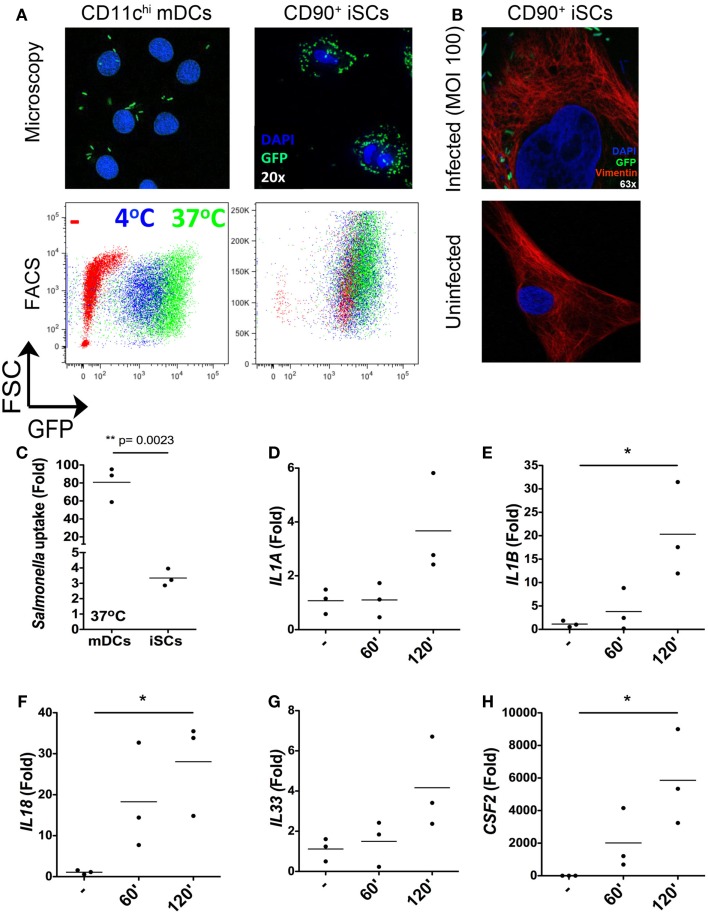
**Rapid internalization and sensing of live *Salmonella* by iSCs**. **(A)** Shows presence of GFP^+^ *Salmonella in* CD11c^+^ mDCs and CD90^+^stromal cells by representative fluorescence microscopy images (upper panels) and flow cytometry plots (lower panels) after 2 h of bacterial exposure. **(B)** (upper panel) shows the presence of GFP-expressing *Salmonella* in Vimentin+ iSCs infected for 4 h at an MOI of 100; uninfected iSCs shown for comparison (lower panel). **(C)** Shows quantification of *Salmonella* uptake/internalization by mDCs and iSCs exposed to bacteria as in **(A)**, expressed as the mean fold change in GFP MFI determined by flow cytometry. **(D–H)** Show the mean fold increases in expression of the indicated genes after exposure of iSCs to *Salmonella* at an MOI of 10 for the indicated time periods, relative to unstimulated cells. **(A,B)** shows representative images and FACS plots, data in **(C)** quantified from mDCs generated from peripheral blood of *n* = 3 healthy donors, compared to iSCs from colonic tissue of *n* = 3 surgical patients. Data in **(D–H)** are from iSCs isolated from colonic tissue of *n* = 3 patients. *p < 0.05, other *p* values as indicated.

As the nature of iSC responses to isolated bacterial components suggested a capacity for the sensing of intact bacteria by these cells, we next assessed the ability of iSCs to respond to the invasive mucosal pathogen *Salmonella typhimurium* (Figure 3). We first compared the capacity for *Salmonella* to be taken up by/invade iSCs, using as a reference point the well-defined capacity for CD11c^hi^ peripheral blood monocyte-derived dendritic cells (mDCs) to respond to *in vitro* infection with this pathogen. Using both fluorescence microscopy and flow cytometry, the uptake of GFP-expressing *Salmonella* by 2 h post-infection could be clearly visualized for both iSCs and mDCs (Figure 3A). We verified the immunofluorescence images using confocal microscopy, in order to determine the presence of intracellular bacteria within iSCs (Figure 3B). GFP-expressing *Salmonella* were readily detectable adjacent to Vimentin filaments within infected iSCs, determined on stained 0.9 μM slices that suggest bacterial internalization. Such bacteria/GFP signals were entirely absent within uninfected iSCs. When assessed by flow cytometry this bacterial internalization was apparent at 4°C and enhanced at 37°C in mDCs, suggesting a contribution of both invasion by the pathogen and active uptake by the cell. Levels of internalization were comparable at both temperatures for iSCs, suggesting that internalization was primarily as a result of bacterial-mediated processes. When the uptake of bacteria was quantified by flow cytometry as the fold increase in GFP expression by viable CD11c^+^ or CD90^+^ cells, mDCs were found to internalize S*almonella* at a significantly higher level than iSCs (80.78 ± 19.37 vs. 3.35 ± 0.56-fold increases in GFP expression for mDCs vs. iSCs, *p* < 0.01 Figure 3C). Therefore *Salmonella* rapidly accesses the intracellular compartment of iSCs although at a significantly lower rate than observed with mDCs.

In light of the rapid *Salmonella* internalization in iSCs, we next sought to determine the early transcriptional response to infection with intact bacteria. Focusing on the cytokines we found to be induced in iSCs by isolated TLR ligands (Figure 2), we determined the changes in expression of cytokine mRNA by iSCs after 60 and 120 min of exposure to live bacteria *in vitro* (Figures 3D–H). *IL1A* expression was slightly enhanced by short-term infection, although only increased over uninfected cells by ∼fourfold at 120 min (Figure 3D). In contrast the expression of *IL1B* and *IL18* was more substantially increased, with significant fold changes in expression of IL-1β mRNA (∼20-fold, *p* < 0.05) and IL-18 mRNA (∼30-fold, *p* < 0.05) at 120 min post-infection (Figures 3E,F). mRNA levels of both cytokines were also increased at 60 min post-infection. *IL33* expression showed a similarly modest change in expression as *IL1A*, with levels of mRNA increasing by ∼fourfold at 2 h post-infection (Figure 3G). The most dramatic changes in cytokine gene expression were for *CSF2*, the gene encoding GM-CSF, with levels increasing by up to 6000-fold at 2 h post-infection (*p* < 0.05 Figure 3H). Taken together, these data suggest that iSCs can respond rapidly to intracellular infection with *Salmonella* via the transcriptional regulation of multiple IL-1 family cytokines and GM-CSF.

### *Salmonella* infection stimulates IL-1β and IL-33 production by iSCs

**Figure 4:**
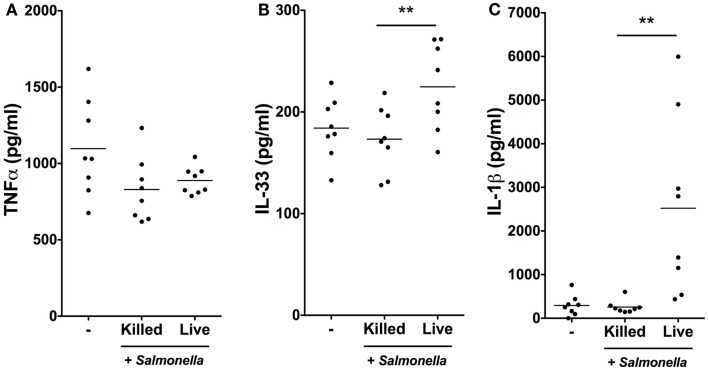
***Salmonella* infection stimulates IL-1β and IL-33 production by iSCs**. **(A–C)** show production levels of the indicated cytokines over a 24 h period by iSCs, determined by ELISA. iSCs were left unstimulated (−) or exposed to live or heat-killed *Salmonella* at an MOI of 10 for 4 h, followed by addition of Gentamycin for the subsequent 20 h. Data are mean levels of cytokine produced by iSCs isolated from colonic tissue of *n* = 8 surgical patients. **p < 0.01.

We next determined whether the rapid transcriptional responses of iSCs to *Salmonella* were associated with corresponding increases in cytokine production at the protein level (Figure 4). We exposed iSCs to live or heat-killed *Salmonella* for 4 h, followed by treatment of cells in all conditions with Gentamycin to kill extracellular bacteria. After 24 h we assessed cell supernatants for levels of iSC cytokine production by ELISA. TNFα was produced constitutively by iSCs, and the levels of this cytokine were not significantly altered by exposure to either heat-killed or live bacteria (Figure 4A). IL-33 was also detectable as a cytokine that was constitutively produced by iSCs (Figure 4B). However in contrast to TNFα, the expression of IL-33 was significantly enhanced upon infection specifically with live *Salmonella*. Exposure to live bacteria significantly increased IL-33 production by iSCs, compared with the response to heat-killed bacteria (224.74 ± 42.80 vs. 173.25 ± 32.24 pg/ml, *p* < 0.01). Exposure to heat-killed bacteria did not significantly modulate levels of iSC IL-33 production from that in the absence of any contact with bacteria. Production of IL-1β by iSCs was dramatically modulated after contact with live *Salmonella* (Figure 4C). This was reflected by an almost 10-fold increase in IL-1β protein production, from 256.84 ± 148.22 pg/ml by iSCs when exposed to heat-killed bacteria to 2522.91 ± 2051 pg/ml (*p* < 0.01) when iSCs were infected with live *Salmonella*. Similar to the results with IL-33, exposure of iSCs to heat-killed bacteria did not increase IL-1β production over the levels observed when iSCs remained unstimulated. Thus iSCs elaborate IL-1β and IL-33 protein in response to infection with live *Salmonella*.

### iSC responses to *Salmonella* require RIPK2/p38MAPK signaling

**Figure 5:**
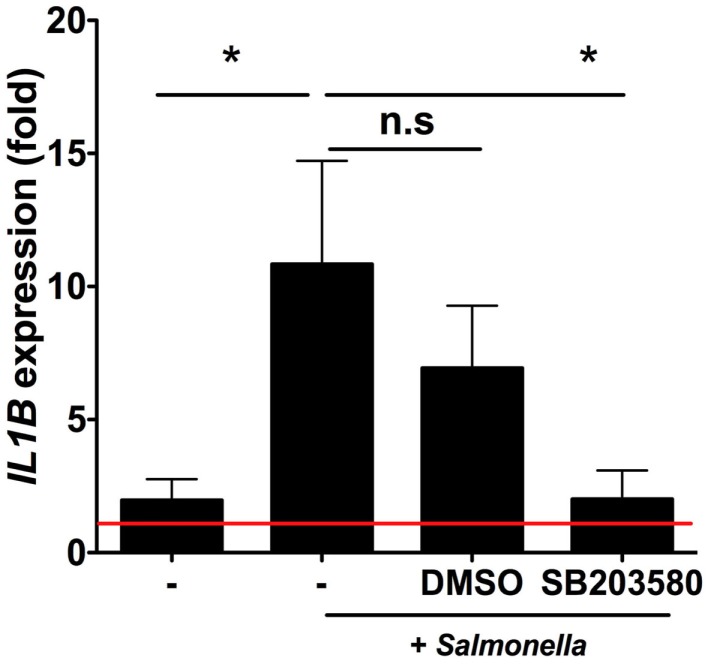
**Intestinal stromal cell responses to *Salmonella* require RIPK2/p38MAPK signaling**. Figure 5 shows relative fold changes in *IL1B* expression determined by qRT-PCR in iSCs left uninfected or after 6 h of infection with live *Salmonella* at an MOI of 10, in the presence of bacteria alone (−), DMSO or SB203580. Data show mean ± SEM fold increases in *IL1B* expression by iSCs under the indicated conditions isolated from *n* = 6 surgical patients. *p < 0.05.

We next sought to investigate the signaling pathways involved in iSC sensing of *Salmonella*. We used the selective RIPK2/p38MAPK inhibitor SB203580 to inhibit signaling pathways downstream of NOD2 (8) (and other PRRs) (Figure 5). Enhanced transcription of *IL1B* in response to *Salmonella* was robustly observed across stromal cells from six individual patients and averaged an ∼10-fold increase over uninfected cells after 6 h of infection (*p* < 0.05). Treatment of iSCs with DMSO alongside *Salmonella* infection had no significant impact on the fold increases in *IL1B* mRNA expression by iSCs. However, treatment with SB203580 completely abrogated the *Salmonella*-induced increase in *IL1B* expression (*p* < 0.05), with an mRNA level indistinguishable from uninfected cells. Thus iSCs require RIPK2/p38MAPK signaling in order to facilitate transcriptional responses to *Salmonella* infection.

### iSCs have a limited capacity for phagocytosis and antigen processing

**Figure 6:**
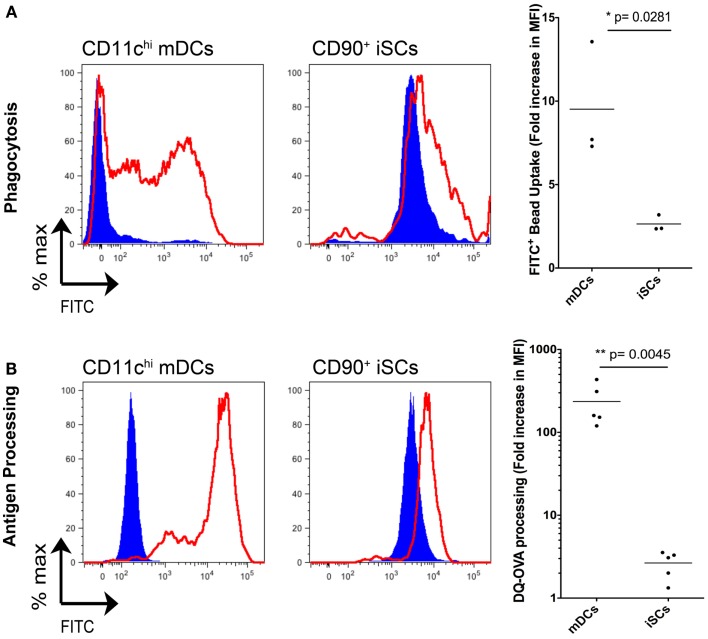
**Intestinal stromal cells have a limited capacity for phagocytosis and antigen processing**. **(A)** shows the quantification of FITC-labeled bead uptake by CD11c^+^ mDCs or CD90^+^ cultured iSCs, determined by flow cytometry at either 4°C (blue filled histograms) or 37°C (red open histograms), expressed as the fold increase in FITC MFI expression by cells at 37°C vs. 4^o^C. **(B)** Shows quantification of antigen processing by CD11c+ mDCs or CD90+ iSCs, determined by flow cytometry after pulsing with DQ-OVA at either 4°C (blue filled histograms) or 37°C (red open histograms), expressed as the fold increase in FITC MFI expression by cells at 37°C vs. 4°C. Data in **(A,B)** show mean fold increases in FITC MFI by mDCs and iSCs from *n* = 3–5 patients. *P* values as indicated.

In light of the capacity for iSCs to sense infection with live bacteria presented above, and in addition their reported expression of HLA-DR (13), we next compared whether iSCs had the capacity to perform functions typically associated with professional innate immune cells/APCs (Figure 6). First we assessed the phagocytic potential of iSCs, again comparing them to CD11c^hi^ mDCs (Figure 6A). After pulsing cells for 4 h at 4°C or 37°C with FITC-labeled latex beads, we determined the phagocytic capacity of mDCs vs. iSCs by assessing the frequencies of FITC positive cells by flow cytometry. At 4°C, mDCs and iSCs showed very low levels of bead uptake that provided the baseline for the assay, with the higher FITC MFI of iSCs a result of the autofluorescent nature of these cells. Pulsing of mDCs at 37°C led to a dramatic uptake of beads by these cells, reflected in an ∼10-fold increase in FITC MFI over background levels. This was in comparison to a significantly lower but still present bead uptake by iSCs, at approximately threefold over cells at 4°C (*p* < 0.05).

We next determined the capacity for iSCs to process antigen. Using FITC expression as a readout, we pulsed mDCs and iSCs for 4 h with DQ-OVA, a fluorescently tagged model antigen with emission quenched until antigen is processed (Figure 6B). Again levels of processing were low in both cell types at 4°C, with no processing detectable at this temperature. At 37°C, the antigen-processing capacity of mDCs and iSCs was strikingly different; mDCs processed antigen efficiently, reflected in an ∼300-fold increase in FITC MFI whereas iSCs were only capable of processing antigen to give an ∼fivefold increase in MFI 4 h after pulsing (*p* < 0.001). Taken together, these data revealed that iSCs have a significantly impaired capacity for both phagocytosis and antigen processing when compared to professional APCs, yet nevertheless retain a limited ability to perform these prototypical innate immune functions.

## Discussion

In this study we show that human iSCs exhibit an array of innate immune functions. Previous studies in the human system had focused mainly on the CCD18co colonic myofibroblastic cell line, and the functional responses upon stromal bacterial challenge were limited to IL-8 expression (3). We have built on these observations with experiments exclusively in primary human colonic stromal cells, and could confirm much of the reported data relating to expression of NOD1, NOD2, and multiple TLRs at the mRNA level. We also provide evidence that broadens the potential range of PRRs expressed by iSCs to include components of the NLR family signaling pathway, including *NLRP1*, *NLRP3*, and the inflammasome adaptor *PYCARD*.

Our functional data validates observations in murine stromal cells that suggested NOD2 signaling could occur in response to *Citrobacter rodentium* infection (4), and reveals for the first time than human iSCs express NOD2 at the protein level and functionally respond with phosphorylation of the signaling intermediate RIPK2 after stimulation with the NOD2 ligand MDP. We found that stimulation with a NOD2 ligand regulated the expression of multiple members of the IL-1 family of cytokines, with NOD2 triggering synergizing with TLR2 stimulation to increase the magnitude of transcriptional responses, a process seemingly conserved between iSCs and myeloid cells (14).

Similar NOD2-dependent innate immune functions in stromal cells have been reported in the human system previously. NOD2 expression is detectable at the mRNA level in oral stromal cells, and MDP stimulation *in vitro* regulates IL-6, IL-8, and CCL2 production by these cells (15). In contrast to the expression of NOD2 mRNA we found in steady-state intestinal SCs, oral stromal cell expression of NOD2 mRNA is dependent on inflammatory cytokines, suggesting differences in basal NOD2 expression by stromal cells dependent on tissue localization. It will be interesting to determine whether there are differences in NLR/PRR expression in stromal cells isolated from distinct regions of the intestinal mucosa, e.g., Ileum vs. Colon, that may contribute to or be affected by the differing bacterial composition at these sites, and the distinct inflammatory lesions that can occur at these anatomical positions during disease.

Similar to stromal cells of the oral mucosa, no basal expression of NOD2 is detectable in Rheumatoid Arthritis Synovial Fibroblasts (RASFs), with NOD2 mRNA expression in these stromal populations only detectable after stimulation with POLYI:C, LPS, or TNFα (16). Interestingly, a similar synergy between NOD2 and TLR2 signaling is observed in cells from this site, whereby co-triggering with ligands of these PRRs synergistically enhances the expression of IL-6 and IL-8 via a p38MAPK-dependent mechanism (16). Synergy in multiple PRR signaling pathways has also been reported (17), and it has been suggested that such synergy may be specific to the TLR2 and NOD2 signaling pathways in stromal cells isolated from the oral mucosa (18, 19). A major outstanding question is whether the signaling pathways underlying the synergy between simultaneous NOD2 and TLR2 triggering are conserved between myeloid and stromal cells or whether cell type-specific pathways result in broadly similar transcriptional and functional outcomes.

We found NOD1 to be highly expressed in iSCs, and indeed this receptor was the only PRR expressed in iSCs at similar levels to monocytes. NOD1 is also strongly expressed in synovial stromal cells and may have a dominant role over NOD2 at this site (20). It is possible that the expression of NOD1 is not as tightly regulated as that of NOD2, reflected in the higher levels of basal expression in stromal cells, further work will be required to address this. Given the role of both NOD1 and NOD2 in directing autophagy as an innate immune strategy in myeloid cells (8, 21), and recent data indicating that human iSCs exhibit similar ATG16L1-dependent autophagosome formation in some contexts (22), it will be interesting to determine whether NOD1 and/or NOD2 can direct autophagy induction as a component of innate defense in the intestinal stromal compartment, in addition to in myeloid cells.

In addition to the responses we observed with NOD2 and TLR2 ligands, we found that iSCs exhibited a particularly strong response to stimulation with extracellular flagellin. Given that extracellular flagellin fails to activate IPAF (NLRC4) in myeloid cells (23) and indeed we found that human iSCs do not express detectable levels of *NLRC4* mRNA, it is unlikely that this NLR mediates the responses we observed. It is thus feasible that flagellin signals via TLR5, which we found expressed by iSCs at the mRNA level, and is also expressed at the basolateral surface of IECs (24). As the basolateral surface of IECs interfaces anatomically with a layer of iSCs in the gut (1), it is likely that both IEC and iSC TLR5 signaling would contribute to the sensing of invasive bacteria that access the subepithelial compartment of the intestine. However, the production of IL-1β by iSCs in response to contact with live, but not heat-killed, *Salmonella* suggests that there may be a requirement for cytosolic sensing of flagellin that may be delivered either by bacterial internalization, which we confirmed was occurring in these cells using confocal microscopy, or via a type III secretion system only operating on a live bacterium. Further work will be required to address this.

In addition to characterizing their innate sensing capacity, we sought to clarify the potential role of iSCs as antigen presenting cells. Previous data indicated that iSCs can express HLA-DR (25), and that this expression is robustly induced in response to IFNγ (13). Previous work had also showed presentation of antigen to a T cell clone following transfection of a specific HLA molecule into the CCD18co cell line, in addition to the capacity for freshly isolated iSCs to induce HLA-DR-dependent IL-2 production by CD4^+^ T cells (13), as well as allowing for SEB-mediated CD4^+^ T cell responses (26). Here we addressed the direct antigen-processing capacity of steady-state iSCs, which indicated that although they have some capacity to process soluble antigen, this function was inferior to that observed in professional APCs. It would be interesting to determine whether this deficit would be less pronounced in iSCs isolated from patients with active inflammation, or by iSCs after stimulation *in vitro* with inflammatory cytokines. It is possible that the lower levels of antigen-processing capacity observed in iSCs may be related to their tolerogenic capacity, as previously indicated by the HLA-DR-dependent induction of regulatory T cells (27) and the functional regulation of CD4^+^ T cell phenotype by iSCs via PD-L1 expression (10). Lymphoid organ stromal cells can mediate T cell tolerance (28), via the presentation of peripheral tissue antigens (29), and can also regulate T cell proliferation via production of nitric oxide (30), although it is not yet clear if similar mechanisms operate in iSCs.

Stromal cells in the gut have a major role in wound healing and epithelial regeneration (31), so in addition to producing chemokines (4) and cytokines in response to bacterial challenge, it is possible that a range of epithelial healing/repair factors are also regulated upon sensing of invasive bacteria. We found that expression of IL-33 was rapidly induced at both the mRNA and protein level upon infection of iSCs with *Salmonella*, and IL-33 may itself be a candidate for a mucosal healing or repair factor, given its role as a major mediator of epithelial regeneration and homeostasis in the gut (32). Interestingly, colonic stromal cells have been reported as major producers of IL-33 during active intestinal inflammation in humans (33) and it is thus possible this enhanced expression may be at least in part a result of the sensing of bacterial translocation by stromal cells at the mucosa during active disease.

An interesting future question is to address whether subsets of iSCs exist *in vivo* with distinct innate immune potential. Previous work has identified massive heterogeneity even within stromal cells isolated from a single human organ (34), and as iSCs have distinct localization and positioning (2), it is feasible that certain iSC subsets are equipped with innate immune functional specialization. Although not yet defined, there is likely to be considerable phenotypic and functional heterogeneity within the iSC compartment, as suggested by recent data characterizing the stromal cell populations of lymph nodes (35, 36). Interestingly, the innate immune capacity of stromal cells appears to be evolutionarily conserved, as stromal cells of teleost fish can act as sentinels and thus sense infection and inflammation (37).

In summary we have shown that human iSCs are able to exhibit multiple innate immune functions, albeit with some of these at significantly limited levels compared with professional innate immune cells. Stromal cells are likely to be important in innate immune responses to invasive pathogens, e.g., invasive *Salmonella*, however it is likely that the epithelium plays a dominant role in innate immunity in the steady-state (38). Nevertheless, after a breach or damage to the contiguous epithelium, or indeed following active invasion by bacteria, stromal cells are perfectly positioned to respond with multiple effector pathways to initiate, coordinate, and focus a professional innate immune response, allowing for both effective defense of the host and for the mucosal barrier to regain homeostasis.

## Conflict of Interest Statement

The authors declare that the research was conducted in the absence of any commercial or financial relationships that could be construed as a potential conflict of interest.
