# Corrigendum: CD90^+^ Stromal Cells are Non-Professional Innate Immune Effectors of the Human Colonic Mucosa

**DOI:** 10.3389/fimmu.2015.00325

**Published:** 2015-06-24

**Authors:** Benjamin M. J. Owens, Tessa A. M. Steevels, Michael Dudek, David Walcott, Mei-Yi Sun, Alice Mayer, Philip Allan, Alison Simmons

**Affiliations:** ^1^Translational Gastroenterology Unit, Nuffield Department of Medicine, Experimental Medicine Division, John Radcliffe Hospital, University of Oxford, Oxford, UK; ^2^MRC Human Immunology Unit, John Radcliffe Hospital, Weatherall Institute of Molecular Medicine, University of Oxford, Oxford, UK

**Keywords:** stromal cells, mucosal immunology, innate immunity, intestinal homeostasis, colon

## Current Acknowledgments

The authors thank Professor David Holden, Imperial College London, for the kind gift of GFP-*Salmonella enterica typhimurium*. The authors thank Dr. Claire Pearson for critical review of the manuscript.

## New, Amended Acknowledgments

The authors thank Professor David Holden, Imperial College London, for the kind gift of GFP-*Salmonella enterica typhimurium*. The authors thank Dr. Claire Pearson for critical review of the manuscript and The Sir Jules Thorn Charitable Trust for their financial support.

## Conflict of Interest Statement

The authors declare that the research was conducted in the absence of any commercial or financial relationships that could be construed as a potential conflict of interest.

